# Long‐term prognosis in breast cancer is associated with residual disease after neoadjuvant systemic therapy but not with initial nodal status

**DOI:** 10.1002/bjs.11963

**Published:** 2021-05-26

**Authors:** L Zetterlund, F Celebioglu, T Hatschek, J Frisell, J de Boniface

**Affiliations:** 1 Department of Clinical Science and Education, Karolinska Institutet, Southern General Hospital Stockholm, Sweden; 2 Department of Surgery, Breast Unit, Capio St Göran's Hospital, Stockholm, Sweden; 3 Department of Surgery, Southern General Hospital, Stockholm, Sweden; 4 Department of Oncology and Pathology, Cancer Centre Karolinska, Karolinska Institutet and University Hospital, Stockholm, Sweden; 5 Department of Molecular Medicine and Surgery, Karolinska Institutet, Stockholm, Sweden

## Abstract

**Background:**

This follow‐up analysis of a Swedish prospective multicentre trial had the primary aim to determine invasive disease‐free (IDFS), breast cancer‐specific (BCSS) and overall survival (OS) rates, and their association with axillary staging results before and after neoadjuvant systemic therapy for breast cancer.

**Methods:**

Women who underwent neoadjuvant systemic therapy for clinically node‐positive (cN+) or ‐negative (cN0) primary breast cancer between 2010 and 2015 were included. Patients had a sentinel lymph node biopsy before and/or after neoadjuvant systemic therapy, and all underwent completion axillary lymph node dissection. Follow‐up was until February 2019. The main outcome measures were IDFS, BCSS and OS. Univariable and multivariable Cox regression analyses were used to identify independent factors associated with survival.

**Results:**

The study included a total of 417 women. Median follow‐up was 48 (range 7–114) months. Nodal status after neoadjuvant systemic therapy, but not before, was significantly associated with crude survival: residual nodal disease (ypN+) resulted in a significantly shorter 5‐year OS compared with a complete nodal response (ypN0) (83·3 *versus* 91·0 per cent; *P =* 0·017). The agreement between breast (ypT) and nodal (ypN) status after neoadjuvant systemic therapy was high, and more so in patients with cN0 tumours (64 of 66, 97 per cent) than those with cN+ disease (49 of 60, 82 per cent) (*P =* 0·005). In multivariable analysis, ypN0 (hazard ratio 0·41, 95 per cent c.i. 0·22 to 0·74; *P =* 0·003) and local radiotherapy (hazard ratio 0·23, 0·08 to 0·64; *P =* 0·005) were associated with improved IDFS, and triple‐negative molecular subtype with worse IDFS.

**Conclusion:**

The present findings underline the prognostic significance of nodal status after neoadjuvant systemic therapy. This confirms the clinical value of surgical axillary staging after neoadjuvant systemic therapy.

## Abstracta

**Antecedentes:**

Se trata de un análisis de seguimiento en el marco de un ensayo multicéntrico prospectivo sueco con el objetivo principal de determinar las tasas de supervivencia libre de enfermedad invasiva (*invasive disease‐free survival*, IDFS), supervivencia específica de cáncer de mama (*breast cáncer‐specific survival*, BCSS) y supervivencia global (*overall survival*, OS) y su asociación con los resultados de la estadificación axilar antes y después de la terapia sistémica neoadyuvante para el cáncer de mama.

**Métodos:**

Se incluyeron un total de 417 mujeres tratadas con NAST por cáncer de mama primario con ganglios clínicamente positivos (cN +) o negativos (cN0) entre 2010 y 2015. Las pacientes tenían una biopsia del ganglio linfático centinela (*sentinel lymph node biopsy*, SLNB) antes y/o después del tratamiento sistémico neoadyuvante y todas se sometieron a una disección completa de los ganglios linfáticos axilares. El seguimiento fue hasta febrero de 2019. Las principales medidas de resultados fueron IDFS, BCSS y OS. Se utilizaron análisis de regresión de Cox uni‐ y multivariables para identificar los factores independientes asociados con la supervivencia.

**Resultados:**

La mediana de seguimiento fue de 48 meses (rango 7‐114). El estado ganglionar después, pero no antes, del tratamiento sistémica neoadyuvante se asoció de forma significativa con la supervivencia cruda: la enfermedad ganglionar residual (ypN +) se asoció con una OS a los 5 años significativamente más corta en comparación con la respuesta ganglionar completa (ypN0: OS 83,3 *versus* 91,0%, *P* = 0,017). La concordancia entre el estado de la mama (ypT) y de los ganglios (ypN) después de la terapia sistémica neoadyuvante fue alta, y más en cN0 (64/66, 97,0%) que en pacientes con cN + (49/60, 81,7%, *P* = 0,005). En el análisis multivariable, ypN0 (cociente de riesgos instantáneos, *hazard ratio*, HR 0,41, i.c. del 95% 0,22‐0,74, *P* = 0,003) y la radioterapia local (HR 0,23 (0,08‐0,64, *P* = 0,005)) se asociaron con un subtipo molecular triple negativo con peor IDFS.

**Conclusión:**

Los presentes hallazgos subrayan la importancia pronóstica del estado ganglionar después de la terapia sistémica neoadyuvante. Esto confirma el valor clínico de la estadificación axilar quirúrgica después de la terapia sistémica neoadyuvante.

This long‐term follow‐up study determined survival rates in a Swedish national cohort of 417 patients with breast cancer who all had neoadjuvant systemic therapy (NAST). Sentinel lymph node biopsy (SLNB) was performed before NAST in clinically node‐negative and after NAST in clinically node‐positive patients. All patients had an axillary lymph node dissection after NAST. Axillary staging results after NAST predicted survival, supporting the practice of SLNB after NAST in all patients. pCR, pathological complete response.

SNLB better after NAST

## Introduction

Neoadjuvant systemic therapy (NAST) was traditionally given to patients with inoperable locally advanced breast cancer, inflammatory breast cancer or when fixed or matted axillary lymph nodes were present. The majority of these women had lymph node‐positive disease at diagnosis^1^, and axillary lymph node dissection (ALND) was performed routinely both as a staging and therapeutic procedure.

NAST is increasingly being offered to women with early breast cancer, especially when aggressive tumour biology has been identified. The axillary lymph nodes are negative in more than half of these patients, but even among those with clinically node‐positive (cN+) disease, an average of 40 per cent have a pathological complete response (pCR) in the nodes after NAST, with specific subgroups such as human epidermal growth factor receptor 2 (HER2)‐positive tumours showing pCR rates as high as 70 per cent^2^^,^^3^. Accordingly, less extensive axillary staging methods, such as sentinel lymph node biopsy (SLNB) or targeted axillary dissection, are implemented in the neoadjuvant setting to spare women the significant morbidity associated with ALND^4^^,^^5^. In patients with clinically node‐negative (cN0) tumours, some debate remains regarding whether SLNB should be performed before or after the completion of NAST. By performing axillary staging after NAST an additional operation is avoided and the downstaging effect of NAST can be evaluated^6^. Zetterlund and colleagues^7^, however, showed that 50 per cent of patients with cN0 disease had a positive SLNB before NAST, with potential implications for adjuvant treatment.

There is significant agreement between nodal pCR (ypN0) and pCR in the breast (ypT0/Tis), especially in cN0 disease^8^^,^^9^. Results from the prospective multicentre GANEA 2 trial^10^ showed that both a residual breast tumour size of at least 5 mm and lymphovascular invasion remained independent predictors of a false‐negative SLNB after NAST in patients with cN+ disease. In an Italian retrospective cohort study^11^, a negative SLNB after NAST was a favourable prognostic factor in all patients except those with residual disease in the breast, thus indicating unidentified residual nodal disease. Even though an increased false‐negative rate of SLNB after NAST has been shown repeatedly^12^, the implication for oncological outcome remains unclear, as there is a lack of follow‐up data on the consequences of omitting ALND after NAST.

The primary aim of the trial from which the present data originate was the validation of SLNB in the neoadjuvant setting and so ALND was performed in all patients. The present analysis is a follow‐up of the Swedish prospective multicentre trial, with the primary aim to determine invasive disease‐free (IDFS), breast cancer‐specific (BCSS) and overall survival (OS) rates and their association with axillary staging results before and after NAST.

## Methods

The Swedish prospective multicentre trial included 419 patients with primary biopsy‐proven invasive breast cancer planned for NAST. All patients underwent completion ALND after NAST. A total of 224 patients with cN0 disease at diagnosis had SLNB before NAST and optionally repeated after NAST^7^. The remaining 195 patients with cytologically confirmed cN+ disease underwent SLNB after completion of NAST^13^. Exclusion criteria were allergy to blue dye or radioactive tracer and inability to provide informed consent. Inflammatory breast cancer was an exclusion criterion for the cN0 arm only.

Patients were recruited from 16 Swedish hospitals between 1 October 2010 and 31 December 2015. Clinical nodal status was determined at diagnosis by clinical examination and axillary ultrasonography. In the event of suspicious lymph nodes, a fine‐needle aspiration biopsy was taken. Surgeons were instructed to perform dual mapping of sentinel lymph nodes with blue dye and isotope; this was done before NAST in 95·5 per cent of women with cN0 disease and after NAST in 87·5 per cent of women with cN+ disease. Nodal status before NAST was divided into three groups: cN0 pN0(sn), cN0 pN+(sn) and cN1. Response evaluation included clinical assessment, imaging and histopathology for breast and lymph nodes separately. Pathological staging (ypTNM) after NAST was based on the seventh edition of the AJCC staging system^14^. A pCR was defined by the absence of residual invasive disease in the breast (ypT0/Tis) or axillary lymph nodes (ypN0). The presence of isolated tumour cells in axillary lymph nodes after NAST was thus not considered nodal pCR^15^^,^^16^. Patients were considered hormone receptor (HR)‐positive if oestrogen receptor (ER)‐ and/or progesterone receptor (PR)‐positive. As expression of the proliferation marker Ki‐67 could not be used reliably owing to uncertainty about historical local cut‐off levels, surrogate molecular tumour subtypes were based solely on immunohistochemical analysis of ER, PR and HER2 status on core needle biopsy at the time of diagnosis. Patients were classified into one of four subgroups: HR+/HER2–, HR–/HER2+, HR+/HER2+ and triple‐negative breast cancer (TNBC). Follow‐up was until February 2019. Follow‐up intervals were not prespecified in the protocol because the present analysis was not planned at the start of the trial.

Local recurrence was defined as a histologically confirmed invasive breast cancer recurrence in the ipsilateral breast or chest wall. Regional recurrence was defined as a recurrence in the ipsilateral or contralateral axillary^17^, ipsilateral infraclavicular or supraclavicular, interpectoral or internal mammary lymph nodes. Distant recurrence was defined as breast cancer recurrence at any other site. Morphological confirmation of distant recurrence was desirable but not mandatory. The site of first recurrence was registered. If a patient presented with concurrent multiple recurrences, any distant recurrence was registered as first recurrence, then any regional and finally any local recurrence.

Adjuvant therapy was not stipulated in the trial protocol. The standard target for locoregional radiotherapy included ipsilateral axillary level 2 and 3 as well as supraclavicular/infraclavicular nodal basins; a few centres routinely included axillary level 1 as well. Parasternal lymph nodes were not routinely included in the radiotherapy target in Sweden during the study interval.

The study was approved by the Regional Ethics Committee at Stockholm County (Dnr 2010/441‐31/4) and the Radiation Protection Committee at Southern General Hospital Stockholm (Dnr 6/10) in 2010, and amended in 2018 (Dnr 2018‐387/32). It was registered at ClinicalTrials.gov (NCT02031042). Written informed consent was obtained from all participants before enrolment.

### Statistical analysis

Categorical variables are reported as numbers with percentages and continuous data as median (range). Pearson's χ^2^ test was used to compare the distribution of categorical variables between cohorts.

The main outcome measures were IDFS, BCSS and OS. IDFS encompasses local, regional, distant and contralateral invasive breast cancer recurrences as well as death from any cause. All *in situ* events were excluded. Data on other primary non‐breast invasive tumours, which were included in the IDFS definition by Hudis and colleagues^18^, were not available. Survival was calculated from the date of diagnosis until the date of any first recurrence or death (IDFS), death from breast cancer (BCSS) or death from any cause (OS). Patients with no events were censored at the date of latest medical chart review.

Five‐year survival rates were calculated by the Kaplan–Meier method, and the log rank test was used to compare groups. To assess the impact of different co‐variables on survival, univariable and multivariable Cox regression analyses were carried out. The clinically most relevant co‐variables were chosen avoiding potentially co‐linear combinations. Cox proportional hazards model assumptions were checked graphically. Patients with any missing data on the chosen co‐variables were excluded from both models. As there were few deaths, multivariable analyses were undertaken only for the outcome IDFS. Co‐variables entered into both univariable and multivariable analyses were age (no more than 40, 41–50, at least 51 years), clinical nodal stage (cN0, cN+), clinical tumour stage (cT1–2, cT3–4), tumour subtypes (HR+/HER2–, HR–/HER2+, HR+/HER2+, TNBC), pCR in the breast (ypT0/Tis; no, yes), pathological complete response in the lymph nodes (ypN0; no, yes) and radiotherapy (none, breast or chest wall, locoregional). Results are presented as hazard ratios with 95 per cent confidence intervals. *P* values from Cox models were based on Wald tests. *P* < 0·050 was considered statistically significant. A test of interaction between all significant variables included in the multivariable regression model was performed. If an interaction effect was identified between two variables, its effect on the outcome was assessed by computing an interaction variable and comparing the hazard ratios for different values of the two interacting variables. Statistical analyses were done using SPSS® version 25 (IBM, Armonk, New York, USA).

## Results

Data from 417 patients were available for follow‐up; two patients from the original trial were excluded from follow‐up analyses as they developed distant metastases during NAST. Median follow‐up after the date of breast cancer diagnosis was 48 (range 7–114) months. The 5‐year OS rate was 87·8 per cent and the BCSS rate was 88·3 per cent. Patient and tumour characteristics as well as neoadjuvant and adjuvant therapy are summarized in *Table* *1*.

**Table 1:** Patient and tumour characteristics and therapy administered to 417 patients included in the follow‐up analysis until February 2019

	No. of patients* (*n* = 417)
**Age (years)** **^†^**	48 (22–84)
**Clinical T status** **^‡^**	
cT1	43 (10·3)
cT2	243 (58·3)
cT3	116 (27·8)
cT4d (inflammatory)	15 (3·6)
**Clinical N status**	
cN0	222 (53·2)
cN+ (biopsy‐verified)	195 (46·8)
**Histological type** **^§^**	
Ductal	337 (80·8)
Lobular	42 (10·1)
Other	30 (7·2)
Missing	8 (1·9)
**Nottingham histological grade** **^§^**	
I	6 (1·4)
II	155 (37·2)
III	141 (33·8)
Missing	115 (27·6)
**Oestrogen receptor status** **^§^**	
Negative	146 (35·0)
Positive	271 (65·0)
**Progesterone receptor status** **^§^**	
Negative	220 (52·8)
Positive	197 (47·2)
**HER2 status** **^§^**	
Not amplified	282 (67·6)
Amplified	134 (32·1)
Missing	1 (0·2)
**Surrogate molecular subtype** **^§^**	
HR+/HER2–	186 (44·6)
HR–/HER2+	50 (12·0)
HR+/HER2+	84 (20·1)
HR–/HER2–	96 (23·0)
Missing	1 (0·2)
**NAST regimen**	
Taxane‐based chemotherapy	270 (64·7)
Taxane + trastuzumab +/– pertuzumab	112 (26·9)
Anthracycline‐based chemotherapy	13 (3·1)
Aromatase inhibitor	3 (0·7)
Other	19 (4·6)
**Breast surgery**	
Breast‐conserving surgery	115 (27·6)
Mastectomy	302 (72·4)
**Adjuvant radiotherapy**	
None	32 (7·7)
Breast/chest wall	69 (16·5)
Locoregional	316 (75·8)
**Adjuvant endocrine therapy**	
Yes (total)	276 (66·2)
Yes (of HR+after NAST)	276 of 281 (98·2)
**Adjuvant anti‐HER2 therapy**	
Yes (total)	139 (33·3)
Yes (of HER2+after NAST)	139 of 139 (100)
**Adjuvant chemotherapy**	66 (15·8)

*With percentages in parentheses unless indicated otherwise;

† values are median (range).

‡ Based on imaging measurements.

§ From core biopsy at time of diagnosis. HER2, human epidermal growth factor receptor 2; HR, hormone receptor; NAST, neoadjuvant systemic therapy.

Sixty‐six of 224 women (29·5 per cent) with cN0 disease initially and 60 of 195 (30·8 per cent) with cN+ disease initially had no residual invasive disease in the breast (ypT0/Tis) after NAST. The proportion of patients with both ypN0 and ypT0/Tis was significantly smaller in cN+ than in cN0 disease (49 of 60 (82 per cent) *versus* 64 of 66 (97 per cent) respectively; *P =* 0·005). The pCR rates in the breast varied significantly between tumour subtypes: 66 per cent for HR–/HER2+, 42 per cent for TNBC, 41 per cent for HR+/HER2+ and 10·1 per cent for HR+/HER2– (*P* < 0·001). The corresponding values for nodal pCR in the cN+ group were 64, 55, 51 and 12·7 per cent respectively (*P* < 0·001).

During follow‐up, 92 of 417 patients (22·1 per cent) experienced recurrence. First events were local in six (7 per cent), regional in 11 (12 per cent), distant in 71 (77 per cent) and contralateral in four (4 per cent) women. Median time to any first recurrence was 26 (range 5–82) months. Of all 92 patients with recurrence, 45 (49 per cent) died from breast cancer during follow‐up. Two patients had non‐breast‐related deaths and no previous recurrences.

Ten of eleven regional recurrences developed in women with cN+ disease at diagnosis. Seven of these had residual nodal disease after NAST and there was one false‐negative SLNB. All seven women eventually developed distant recurrence, resulting in three deaths during follow‐up. In addition, one patient without residual nodal disease after NAST developed distant recurrence.

**Fig. 1:**
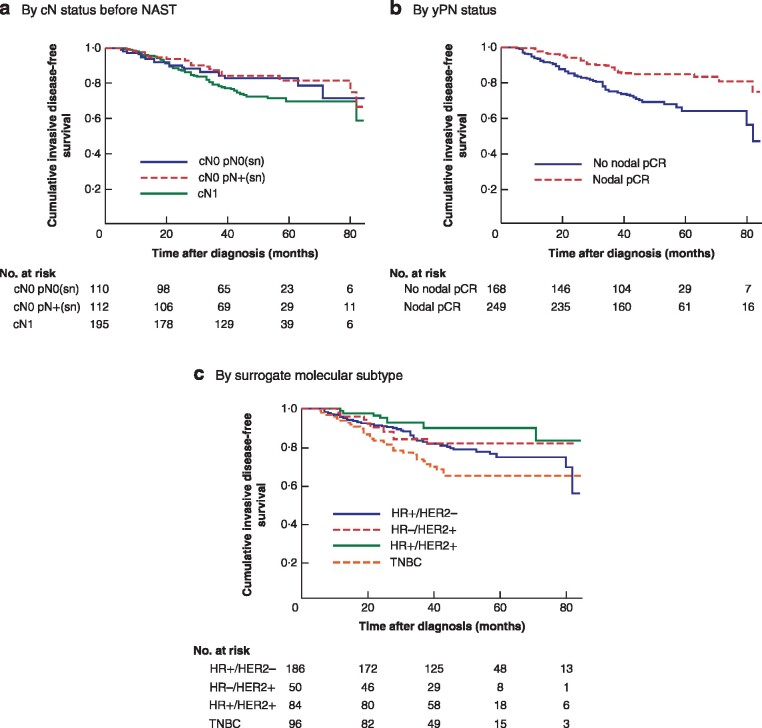
Kaplan–Meier survival curves for invasive disease‐free survival Invasive disease‐free survival according to **a** cN status before neoadjuvant systemic therapy (NAST) (417 patients, 94 events), **b** ypN status after NAST (417 patients, 94 events) and **c** surrogate molecular tumour subtype (416 patients, 93 events). sn, sentinel node; pCR, pathological complete response; HR, hormone receptor; HER2, human epidermal growth factor receptor 2; TNBC, triple‐negative breast cancer. **a** *P* = 0·078, **b** *P* < 0·001, **c** *P* = 0·003 (log rank test).

Unadjusted survival curves for IDFS according to clinical nodal status before NAST (cN), pathological nodal status after NAST (ypN) and surrogate molecular subtype are shown in *Fig*. *1*. In contrast to cN status, ypN status was significantly associated with all survival rates including IDFS, BCSS and OS. The 5‐year OS rate was 90·0 *versus* 85·5 per cent for cN0 *versus* cN+ (*P =* 0·198), but 83·3 *versus* 91·0 per cent for ypN+ *versus* ypN0 (*P =* 0·017).

Surrogate molecular tumour subtype was a strong prognostic factor; unadjusted 5‐year BCSS rates were 73 per cent in TNBC, 87 per cent in HR–/HER2+, 90·6 per cent in HR+/HER2– and 100 per cent in HR+/HER2+ subtypes (*P* < 0·001). Corresponding rates for 5‐year OS were 73, 87, 89·6 and 100 per cent (*P* < 0·001).

There were 94 events, and 45 and 47 deaths among 417 women for the survival outcomes IDFS, BCSS and OS respectively. One patient was excluded from analyses because a value was missing for one of the co‐variables. In multivariable Cox regression analysis, pCR in the axillary lymph nodes (hazard ratio 0·41, 95 per cent c.i. 0·22 to 0·74; *P =* 0·003) and adjuvant radiotherapy to the breast or chest wall (hazard ratio 0·23, 0·08 to 0·64; *P =* 0·005) were independently associated with improved IDFS, whereas TNBC subtype (hazard ratio 2·87, 1·72 to 4·79; *P* < 0·001) had an independent association with worse survival (*Table* *2*). There was a statistically significant interaction effect between ypN and adjuvant radiotherapy for the outcome IDFS. The hazard ratio for radiotherapy was further decreased when nodal pCR was achieved.

**Table 2:** Univariable and multivariable Cox regression analyses for invasive disease‐free survival

	No. of patients (*n* = 416)	No. of events (*n* = 93)	Univariable analysis		Multivariable analysis	
			Hazard ratio*	*P*	Hazard ratio*	*P*
**Age (years)**				0·669		0·338
≤ 40	111	28 (25·2)	1·00 (reference)		1·00 (reference)	
41–50	118	27 (22·9)	0·86 (0·51, 1·46)	0·571	0·73 (0·43, 1·26)	0·260
≥ 51	187	38 (20·3)	0·80 (0·49, 1·31)	0·373	0·70 (0·42, 1·15)	0·160
**cN status**						
cN0	221	39 (17·6)	1·00 (reference)		1·00 (reference)	
cN+	195	54 (27·7)	1·62 (1·07, 2·45)	0·021	1·09 (0·64, 1·86)	0·758
**cT status**						
cT1–2	285	61 (21·4)	1·00 (reference)		1·00 (reference)	
cT3–4d	131	32 (24·4)	1·06 (0·74, 1·71)	0·797	0·95 (0·61, 1·47)	0·806
**Tumour subtype**				0·005		< 0·001
HR+/HER2–	186	44 (23·7)	1·00 (reference)		1·00 (reference)	
HR–/HER2+	50	9 (18)	0·81 (0·40, 1·67)	0·572	1·64 (0·76, 3·53)	0·207
HR+/HER2+	84	9 (11)	0·44 (0·22, 0·91)	0·026	0·56 (0·27, 1·17)	0·125
TNBC	96	31 (32)	1·59 (1·00, 2·52)	0·049	2·87 (1·72, 4·79)	< 0·001
**ypT0/Tis**						
No	290	77 (26·6)	1·00 (reference)		1·00 (reference)	
Yes	126	16 (12·7)	0·47 (0·28, 0·81)	0·006	0·54 (0·29, 1·00)	0·052
**ypN0**						
No	168	55 (32·7)	1·00 (reference)		1·00 (reference)	
Yes	248	38 (15·3)	0·45 (0·30, 0·68)	< 0·001	0·41 (0·22, 0·74)	0·003
**Radiotherapy**				0·037		0·019
No	32	9 (28)	1·00 (reference)		1·00 (reference)	
Breast/chest wall	68	6 (9)	0·28 (0·10, 0·79)	0·016	0·23 (0·08, 0·64)	0·005
Locoregional	316	78 (24·7)	0·78 (0·39, 1·55)	0·472	0·48 (0·21, 1·09)	0·079

Values in parentheses are percentages unless indicated otherwise;

*values are 95 per cent confidence intervals. Includes patients with data available for all co‐variables; analyses are based on 416 patients and 93 events owing to missing information on human epidermal growth factor receptor 2 (HER2) status for one patient. HR, hormone receptor; TNBC, triple‐negative breast cancer; ypT0/Tis, no residual invasive tumour in the breast; ypN0, no residual axillary disease.

In univariable Cox regression analysis, nodal pCR and HR+/HER2+ subtype were associated with improved BCSS and OS, whereas TNBC subtype was associated with both worse BCSS and OS (*Table* *3*).

**Table 3:** Univariable Cox regression analyses for breast cancer‐specific and overall survival

	No. of patients (*n* = 416)	Breast cancer‐specific survival			Overall survival		
		No. of deaths (*n* = 44)	Hazard ratio*	*P*	No. of deaths (*n* = 46)	Hazard ratio*	*P*
**Age (years)**				0·917			0·931
≤ 40	111	12 (10·8)	1·00 (reference)		12 (10·8)	1·00 (reference)	
41–50	118	12 (10·2)	0·91 (0·41, 2·04)	0·822	13 (11·0)	0·99 (0·45, 2·18)	0·979
≥ 51	187	20 (10·7)	1·06 (0·52, 2·04)	0·870	21 (11·2)	1·11 (0·55, 2·27)	0·770
**cN status**							
cN0	221	18 (8·1)	1·00 (reference)		20 (9·0)	1·00 (reference)	
cN+	195	26 (13·3)	1·61 (0·88, 2·96)	0·121	26 (13·3)	1·45 (0·81, 2·61)	0·215
**cT status**							
cT1–2	285	29 (10·2)	1·00 (reference)		30 (10·5)	1·00 (reference)	
cT3–4d	131	15 (11·5)	1·08 (0·59, 1·97)	0·883	16 (12·2)	0·99 (0·54, 1·82)	0·975
**Tumour subtype**				0·002			0·003
HR+/HER2–	186	18 (9·7)	1·00 (reference)		20 (10·8)	1·00 (reference)	
HR–/HER2+	50	5 (10)	1·15 (0·42, 3·10)	0·785	5 (10)	1·03 (0·38, 2·74)	0·959
HR+/HER2+	84	1 (1)	0·13 (0·02, 0·95)	0·044	1 (1)	0·11 (0·02, 0·85)	0·034
TNBC	96	20 (21)	2·60 (1·37, 4·94)	0·004	20 (21)	2·32 (1·24, 4·33)	0·008
**ypT0/Tis**							
No	290	37 (12·8)	1·00 (reference)		39 (13·4)	1·00 (reference)	
Yes	126	7 (5·6)	0·45 (0·20, 1·02)	0·056	7 (5·6)	0·43 (0·19, 0·96)	0·040
**ypN0**							
No	168	25 (14·9)	1·00 (reference)		26 (15·5)	1·00 (reference)	
Yes	248	19 (7·7)	0·50 (0·27, 0·91)	0·022	20 (8·1)	0·50 (0·28, 0·91)	0·022
**Radiotherapy**				0·078			0·110
No	32	1 (3)	1·00 (reference)		2 (6)	1·00 (reference)	
Breast/chest wall	68	2 (3)	0·83 (0·07, 9·19)	0·879	2 (3)	0·42 (0·06, 2·97)	0·382
Locoregional	316	41 (13·0)	3·41 (0·47, 24·83)	0·226	42 (13·3)	1·76 (0·42, 7·28)	0·436

Values in parentheses are percentages unless indicated otherwise;

*values are 95 per cent confidence intervals. Includes patients with data available for all co‐variables; breast cancer‐specific and overall survival analyses are based on 44 and 46 deaths respectively in 416 patients owing to missing information on tumour subtype (HER2 status) in one patient. HR, hormone receptor; HER2, human epidermal growth factor receptor 2; TNBC, triple‐negative breast cancer; ypT0/Tis, no residual invasive tumour in the breast; ypN0, no residual axillary disease.

## Discussion

This follow‐up analysis based on data from a Swedish prospective multicentre trial evaluating SLNB in the neoadjuvant setting showed a lack of association between nodal status before NAST and survival outcomes. Instead, axillary staging results after NAST predicted survival, thus confirming the prognostic importance of axillary status after NAST and supporting the practice of surgical axillary staging after NAST in all patients.

In patients with cN0 disease, SLNB is increasingly being performed after completion of NAST worldwide. This choice of timing takes advantage of the downstaging effect of NAST and an extra surgical procedure is avoided before NAST^6^. In Sweden, however, until recently the recommended procedure has been SLNB before NAST, as it is perceived that nodal staging before NAST allows locoregional treatment decisions to be made without the risk of undertreatment; this is further underlined by the sensitivity of axillary ultrasonography and needle biopsy in patients with suspicious lymph nodes being only approximately 25 per cent^19^. In addition, SLNB identification rates are somewhat lower and false‐negative rates higher after NAST in patients with cN0 disease, but even more so in those with cN+ status^20^. Still, at the 2017 St Gallen conference, only 20 per cent supported SLNB before NAST in patients with clinically node‐negative disease^21^.

In 2012, Mamounas and colleagues^22^ reported predictors of locoregional recurrence based on a combined analysis of the National Surgical Adjuvant Breast and Bowel Project B‐18 and B‐27 trials and concluded that, in addition to age and clinical stage at diagnosis, pathological nodal status and breast tumour response after NAST were the predictors with the greatest impact on locoregional recurrence rates. Of note, these trials did not allow chest wall radiotherapy after mastectomy or regional nodal radiotherapy. In the present study, nodal status before NAST was not associated with survival, but residual nodal disease after NAST was, supporting findings reported by Kuerer and co‐workers in 1999^23^. In 2002, Rouzier *et al*.^24^ suggested axillary status after NAST to be of greater prognostic significance than the response of the primary tumour itself^24^. In the present analysis, ypN0 was not an independent prognostic factor for BCSS and OS; however, it was independently associated with improved IDFS, as was local radiotherapy. Earlier studies also demonstrated the importance of radiotherapy for locoregional control, but without a significant effect on survival^25^. In the present trial, radiotherapy after NAST was not stipulated in the protocol, and the results should be regarded in the light of this limitation. Patients eligible for NAST, however, can be assumed to be suitable for guideline‐compliant radiotherapy also if they have not received radiotherapy previously.

In 2016, Galimberti and colleagues^11^ reported equal 5‐year OS rates in patients with cN1/N2 disease and those with cN0 tumours if they were clinically node‐negative (ycN0) and SLNB‐negative (ypN0(sn)) after NAST. A negative SLNB after NAST, however, was not a favourable prognostic factor in patients with residual disease in the breast, which potentially indicates a higher likelihood of a false‐negative SLNB in this group^11^. Zetterlund and co‐workers^7^^,^^13^ showed that a complete or near‐complete pathological response in the breast was significantly less common in patients with a false‐negative SLNB, regardless of whether SLNB was performed before or after NAST.

Several studies have recently presented data showing a significant agreement between response rates in the breast and axillary lymph nodes, especially in patients with initial cN0 status^8^^,^^9^. This association was confirmed in the present study. Tadros *et al*.^9^ concluded that the risk of missing nodal metastases if surgical axillary staging is omitted is very small in patients with initial cN0 status and no residual disease in the breast, especially in the triple‐negative and HER2‐positive subgroups. In the light of de‐escalated surgery, risk scoring systems are emerging with the aim of identifying women with the highest likelihood of nodal conversion who might be spared any axillary surgery after NAST^26^. In patients with cN0 disease and a negative SLNB after NAST, most women can now be spared an ALND. In those with biopsy‐proven cN+ status, SLNB may be performed after completion of NAST, but false‐negative rates have been unacceptably high^27^. Efforts have been made to decrease the false‐negative rates by selecting patients with ycN0 status after NAST, performing dual mapping, trying to remove at least two sentinel nodes, and marking the positive lymph nodes before NAST and removing them in combination with SLNB after NAST (targeted axillary dissection)^4^.

As all participants in the present trial underwent completion ALND after NAST, the prognostic impact of de‐escalated axillary staging in the neoadjuvant setting could not be evaluated and was not the aim of the analysis. More data can be expected from two ongoing RCTs investigating de‐escalation in axillary treatment after NAST in patients with cN+ disease^28^.

This long‐term follow‐up study of a prospective national cohort of patients with breast cancer who had NAST supports axillary staging after NAST in all patients, and has confirmed the significant association between pCR in the breast and axillary lymph nodes, especially in women with initial cN0 status.

## Notes

Presented to the European Breast Cancer Conference 12, Barcelona, Spain, September 2020; published in abstract form in published in *Eur J Cancer*
